# Proteomic Analysis of Cattle Tick *Rhipicephalus* (*Boophilus*) *microplus* Saliva: A Comparison between Partially and Fully Engorged Females

**DOI:** 10.1371/journal.pone.0094831

**Published:** 2014-04-24

**Authors:** Lucas Tirloni, José Reck, Renata Maria Soares Terra, João Ricardo Martins, Albert Mulenga, Nicholas E. Sherman, Jay W. Fox, John R. Yates, Carlos Termignoni, Antônio F. M. Pinto, Itabajara da Silva Vaz

**Affiliations:** 1 Centro de Biotecnologia, Universidade Federal do Rio Grande do Sul, Porto Alegre, RS, Brazil; 2 Department of Entomology, Texas A&M University, College Station, Texas, United States of America; 3 Instituto de Pesquisas Veterinárias Desidério Finamor, Fundação Estadual de Pesquisa Agropecuária, Eldorado do Sul, RS, Brazil; 4 Department of Microbiology, University of Virginia, Charlottesville, Virginia, United States of America; 5 CAPES, Ministério da Educação do Brasil, Brasília, DF, Brasil; 6 Departamento de Bioquímica, Universidade Federal do Rio Grande do Sul, Porto Alegre, RS, Brazil; 7 Faculdade de Veterinária, Universidade Federal do Rio Grande do Sul, Porto Alegre, RS, Brazil; 8 Department of Chemical Physiology, The Scripps Research Institute, La Jolla, California, United States of America; University of Tours, France

## Abstract

The cattle tick *Rhipicephalus* (*Boophilus*) *microplus* is one of the most harmful parasites affecting bovines. Similarly to other hematophagous ectoparasites, *R. microplus* saliva contains a collection of bioactive compounds that inhibit host defenses against tick feeding activity. Thus, the study of tick salivary components offers opportunities for the development of immunological based tick control methods and medicinal applications. So far, only a few proteins have been identified in cattle tick saliva. The aim of this work was to identify proteins present in *R. microplus* female tick saliva at different feeding stages. Proteomic analysis of *R. microplus* saliva allowed identifying peptides corresponding to 187 and 68 tick and bovine proteins, respectively. Our data confirm that (i) *R. microplus* saliva is complex, and (ii) that there are remarkable differences in saliva composition between partially engorged and fully engorged female ticks. *R. microplus* saliva is rich mainly in (i) hemelipoproteins and other transporter proteins, (ii) secreted cross-tick species conserved proteins, (iii) lipocalins, (iv) peptidase inhibitors, (v) antimicrobial peptides, (vii) glycine-rich proteins, (viii) housekeeping proteins and (ix) host proteins. This investigation represents the first proteomic study about *R. microplus* saliva, and reports the most comprehensive Ixodidae tick saliva proteome published to date. Our results improve the understanding of tick salivary modulators of host defense to tick feeding, and provide novel information on the tick-host relationship.

## Introduction

The cattle tick *Rhipicephalus* (*Boophilus*) *microplus* is a one-host tick that feeds on bovines. It is considered one of the most harmful cattle parasites in sub-tropical areas of the world due to its economic importance [1]. The economic losses associated with *R. microplus* parasitism are (i) direct, i.e., blood loss and lesions that predispose animals to myiasis and anaemia, reducing weight gain and milk production, and (ii) indirect, via the transmission of tick-borne pathogens such as *Babesia* spp. and *Anaplasma marginale* [2], [3].

Like all hematophagous parasites, *R. microplus* salivary secretion is a complex mixture, rich in bioactive compounds that modulate host defenses to tick feeding activity [4]–[7]. In recent decades, transcriptomic and proteomic analyses of salivary glands (sialomes) of several ticks have provided a better insight into the immunobiology at the tick–host interface [4], [5], [7]–[16]. However, in comparison with other hematophagous arthropods, much has yet to be established about the components of *R. microplus* saliva, particularly taking into account the considerable economic losses this parasite causes. *Amblyomma americanum*, *Ixodes scapularis*, *Ornithodoros moubata* and *Rhipicephalus sanguineus* are the only tick species whose saliva has been the object of proteomic analysis [17]–[20]. To date, no comprehensive analysis of *R. microplus* tick salivary proteins has been performed.

There is evidence that tick salivary protein profiles change during tick feeding [21]–[23]. However, it is unclear whether the compounds secreted through *R. microplus* saliva vary throughout tick lifecycle. The identification of tick bioactive salivary components may be a potentially useful tool to more fully understand tick modulation of host physiological system. Moreover, this information may become valuable in the potential identification of novel target antigens for the development of anti-*R. microplus* vaccines and of potential lead compounds for pharmacological applications [24], [25]. The aim of this work was to identify proteins secreted in saliva of *R. microplus* female ticks at two different feeding stages, and to gain insight into the putative role(s) these proteins play in regulating the tick-host relationship. For this purpose, we performed a proteomic characterization of saliva from partially engorged and fully engorged *R. microplus* tick females.

## Materials and Methods

### Ethics statement

All animals used in these experiments were housed in Faculdade de Veterinária, Universidade Federal do Rio Grande do Sul (UFRGS). This study was conducted considering ethic and methodological aspects in agreement with the International and National Directives and Norms by the Animal Experimentation Ethics Committee of the Universidade Federal do Rio Grande do Sul (UFRGS). The protocol was approved by the Comissão de Ética no Uso de Animais (CEUA) - UFRGS.

### Ticks

*R. microplus* ticks, Porto Alegre strain, free of pathogens such as *Babesia* spp. and *Anaplasma* spp. were obtained from a laboratory colony maintained as previously described [26]. Ticks used in this study were exclusively fed on Hereford calves (*Bos taurus taurus*) acquired from a tick-free area. The calves were infested with 10-day-old *R. microplus* larvae.

### Saliva collection

Fully engorged female (FEF) ticks were obtained after the spontaneous detachment from the calves. Partially engorged female (PEF) ticks were carefully detached from the calves' skin by hand, between the 17^th^ and 20^th^ days post-infestation. Mean length of PEF and FEF ticks was 4.5 mm (ranging from 4 to 5 mm) and 11 mm (ranging from 9 to 12.5 mm), respectively. Before saliva collection, any host contaminating tissue in tick mouthparts was removed using a scalpel blade and surgical forceps. PEF and FEF ticks were rinsed with sterile distilled water and induced to salivate by dorsal injection of 2 or 5 µL pilocarpine (2% in PBS), respectively [27], [28]. The saliva accumulated in the mouthparts was periodically collected using a pipette tip from ticks maintained at 37°C in a humid chamber for approximately 3 h. The saliva was stored at −80°C upon use. Saliva protein concentration was determined according to the bicinchoninic acid method (BCA Protein Assay, Pierce, Rockford, USA), as previously described [29].

### *In solution* digestion, liquid chromatography and tandem mass spectrometry (LC-MS/MS) analysis

Three micrograms of protein from PEF and FEF tick saliva were reduced (10 mM DTT), alkylated (50 mM iodoacetamide) and digested with 1 µg modified trypsin (Promega Co., Madison, WI, USA) overnight at room temperature. LC-MS/MS was performed using a Thermo Electron LTQFT hybrid linear ion trap-FTICR mass spectrometer. Samples were loaded into a capillary C18 column (75 µm×7.5 cm) and injected into the mass spectrometer at approximately 500 nL/min. The gradient elution was 0–90% acetonitrile/0.1 M acetic acid over 2 h. Data was collected in a top 10 mode, meaning that one FT scan (100 K resolution) taken was followed by 10 MS/MS fragmentation spectra of the top intensity ions collected in the linear ion trap. After MS/MS fragmentation was performed on a particular parent ion, m/z was placed on an exclusion list to enable greater dynamic range and prevent repeated analysis of the same peptide. Electrospray voltage was set to 2.5 kV, and capillary temperature was 210°C.

Protein and peptide identification and protein quantitation were carried out in an Integrated Proteomics Pipeline - IP2 (Integrated Proteomics Applications, Inc., San Diego, CA, http://www.integratedproteomics.com/). Mass spectra were extracted from raw files using RawExtract 1.9.9.2 [30] and searched against a local *R. microplus* protein database (Rm-INCT-EM) containing 22,009 sequences produced by our research group using Illumina Sequencing technology (BioProject ID PRJNA232001 at Transcriptome Shotgun Assembly (TSA) database – GenBank) with reversed sequences using ProLuCID [31], [32]. Additionally, a bovine protein database (IPI *Bos taurus* -ftp://ftp.ebi.ac.uk/pub/databases/IPI/last_release/current/ipi.BOVIN.fasta.gz) was used to identify host proteins. The search space included all fully-tryptic and half-tryptic peptide candidates. Carbamidomethylation of cysteine was considered as differential modification. Peptide candidates were filtered using DTASelect, with the parameters -p 2 -y 1 -trypstat -pfp .01 –dm [30], [33].

### 1D gel electrophoresis and LC-MS/MS (1D-LC-MS/MS)

Saliva samples (25 µg) of both PEF and FEF were electrophoresed in 12% SDS-PAGE and stained with Coomassie brilliant blue. Subsequently, stained gel band slices (42 to PEF and 15 to FEF) were excised and individually subjected to trypsin digestion, as previously described [34]. The resulting peptides were analyzed using an electrospray ionization (ESI) quadrupole time-of-flight (Q-TOF) MicroTM mass spectrometer (Waters, Milford, MA, USA) coupled to a capillary liquid chromatography system nanoACQUITY UPLC (Waters, Milford, MA, USA). The peptides were eluted from a reverse-phase C18 column toward the mass spectrometer. Charged peptide ions (+2 and +3) were automatically mass selected and dissociated in MS/MS experiments. MS/MS spectra were searched against the database described above (item 2.3) using the MASCOT software version 2.2 (Matrix Science, London, UK) with the following parameters: tryptic specificity, one missed cleavage and a mass measurement tolerance of 0.2 Da in the MS mode and 0.2 Da for MS/MS ions. The carbamidomethylation of cysteine was set as a fixed modification, and methionine oxidation was set as variable modifications. The Scaffold software version 4.0.5 (Proteome Software Inc., Portland, OR) was used to validate MS/MS based peptide and protein identifications. Peptide identifications were accepted if they exceeded specific database search engine thresholds. Mascot identifications required ion scores higher than the associated identity scores of 20 and 35 for doubly and triply charged peptides, respectively. Protein identifications were accepted if they contained at least 2 identified peptides. To be included in this analysis, all peptide sequences had to have 100% identity with assigned proteins.

### Functional annotation and classification of proteins

For functional annotation of the proteins, BLAST tools were used to compare the protein sequences to the NCBI (http://www.ncbi.nlm.nih.gov/) and GeneOntology protein database [35]. The ScanProsite and Pfam servers were used to search for conserved protein domains [36], [37]. Functional annotation of identified tick proteins was based on previously published tick sialomes with some modifications (immunoglobulin-binding proteins were added to this classification) [4].

## Results and Discussion

Blood is the only form of nutrition taken by ticks, and large blood meals are required for their development and survival. Ticks are pool feeders that accomplish feeding by lacerating small blood vessels and sucking up the blood that flows to the wound, the so-called feeding site [4]–[7]. Within minutes of inserting the hypostome into host skin, ticks secrete an amorphous adhesive substance (cement) that anchors them onto host skin and secures attachment throughout the feeding period [38]. When completely attached to the wound site, most ticks slowly feed off the pooled blood at the feeding site for several days [39]. The tick feeding cycle includes (i) the preparatory feeding phase, when the tick attaches onto host skin and creates the feeding lesion; (ii) the slow feeding phase, when the tick swallows moderate amounts of blood, begins to transmit pathogens, and grows new tissue to prepare itself for (iii) the rapid feeding phase, when it feeds to repletion [38], [39]. The tick feeding style triggers tissue repair and other defense responses, like hemostasis, inflammatory reactions, pain or itching, and immune rejection [4]–[7]. Like other blood-sucking parasites, *R. microplus* ticks have developed a complex and sophisticated collection of pharmacological bioactive proteins and lipids produced by salivary glands that counteract host defenses and allow successful parasitism [4], [5]. During blood meal acquisition, salivary glands undergo remarkable growth and differentiation accompanied by significant increase in protein synthesis [21]–[23]. Ticks concentrate the blood meal by secreting excess water and ions back into the host through salivary secretion [40]. After detachment from the host, a signal triggers tick salivary gland degeneration [41], [42]. *R. microplus* ticks attach to its host as unfed larvae, and then proceed to feed and molt through nymphal and immature adult stages in a period that stretches to 12 days. After mating, adult pre-engorged females (PEF) increase blood meal ingestion rapidly, and by the 21^st^ or 22^nd^ day these fully engorged females (FEF) complete feeding and detach [43], [44]. Adult ticks used in this study were collected between days 17 and 22 after experimental infestation. Thus, data presented here represent part of the slow feeding phase and of the final rapid feeding phase. Consistent with reports that other tick species change salivary expression profiles during feeding [21]–[23], data in this study reveals remarkable, quantitative and qualitative differences in saliva content of *R. microplus* at different feeding stages, suggesting modulation of protein expression during these stages. The saliva collection procedure yielded approximately 0.1 µL *per* PEF tick, and on average 0.8 µL of saliva *per* FEF tick. Despite the low amount of saliva secreted by PEF ticks using the pilocarpine-induced method, their salivary secretion had a higher protein concentration (3.22 µg/µL), compared with those obtained from FEF ticks (1.75 µg/µL). This is in accordance with an increased expression of saliva proteins that are important in hematophagy, during slow feeding phase (PEF). Most of these proteins may have been turned off in FEF. This could also be explained by fast degeneration of salivary glands in FEF ticks immediately after detaching from the host [41], [42]. In the same way, as the salivary gland is responsible for hydrodynamic equilibrium in ticks [45] it is supposed that it excretes more water in the rapid feeding phase (FEF) than in the slow feeding phase (PEF), so the volume of saliva is higher in FEF, however protein concentration is lower. The proteomic analysis of *R. microplus* saliva allowed identifying 187 and 68 proteins from tick and cattle, respectively. Sequences from tick identified proteins were deposited as Transcriptome Shotgun Assembly project at DDBJ/EMBL/GenBank under the accessions GBBO00000000 and GBBR00000000. The versions described in this paper are the first version, GBBO01000000 and GBBR01000000, respectively

**Figure 1:**
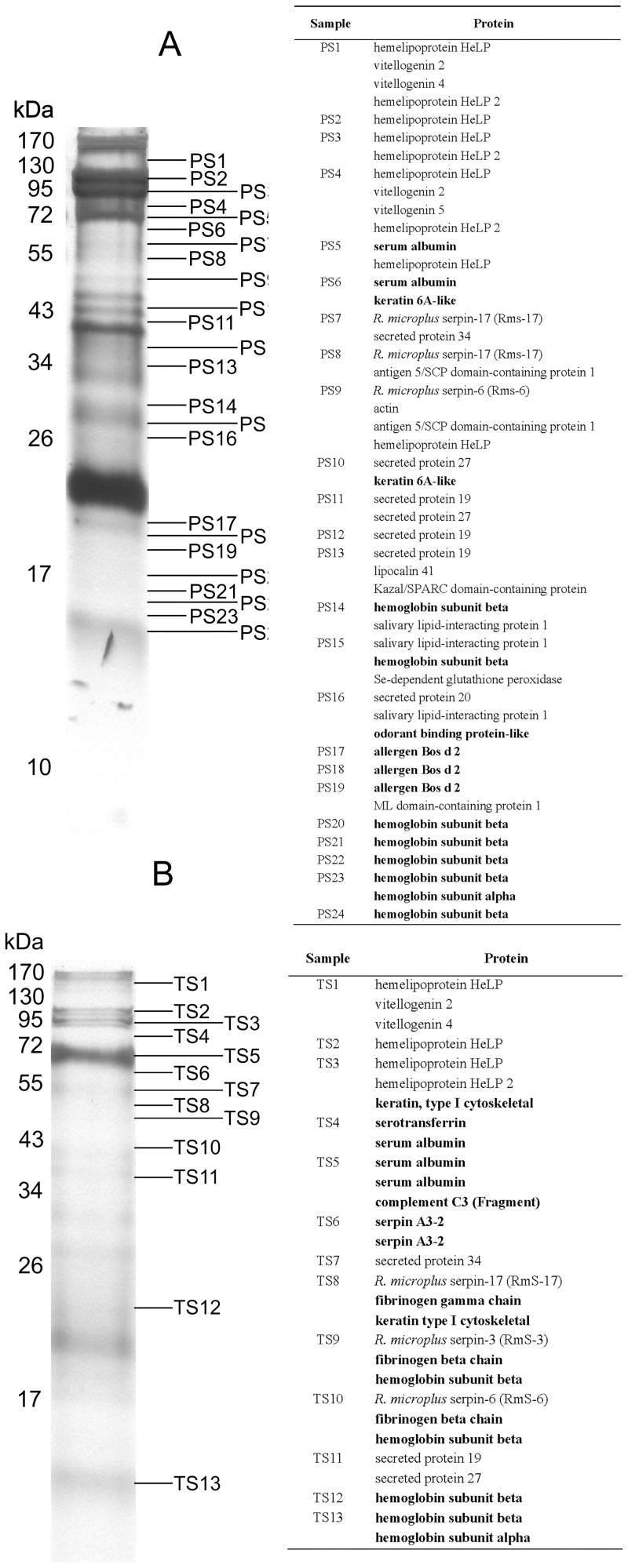
Proteome of *R. microplus* saliva. Saliva (25 µg) from partially engorged females (PEF) (A) and fully engorged females ticks (FEF) (B) was electrophoresed in 12% SDS-PAGE. The bands were excised, submitted for tryptic digestion and identified by LC–MS/MS. Numbers at the left indicate the MW in kDa of the protein standards. Host proteins identified are presented in bold. For further description of protein identification see Table S1 and Table S2.

**Figure 2:**
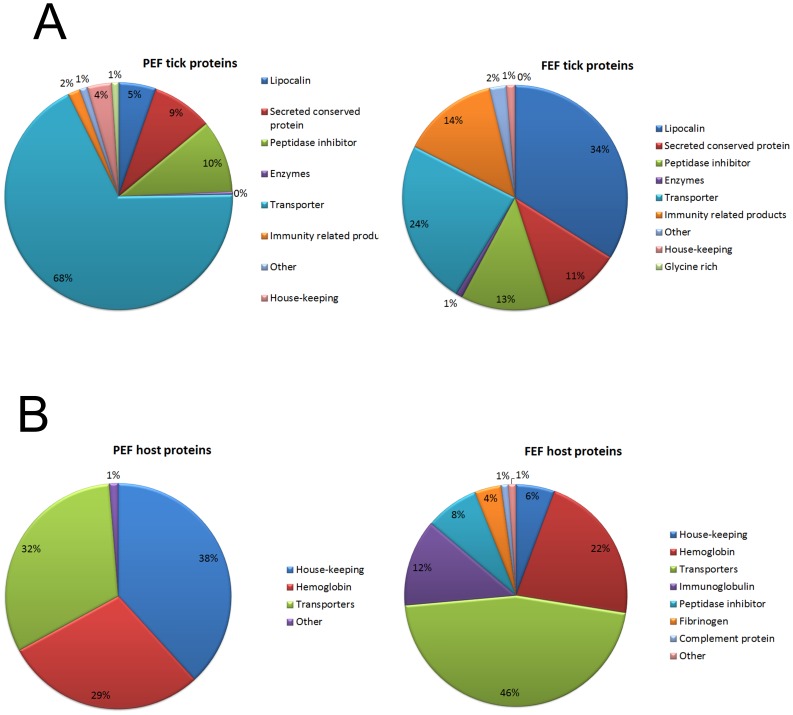
Functional classification of proteins in *R. microplus* saliva. Tick proteins (A) and host proteins (B) identified in *R. microplus* saliva were classified as putative secreted proteins or putative housekeeping proteins, and further in groups according to their function and/or protein family. Pie charts represent the percentage of proteins found in each group with respect to normalized spectral count (in brackets).

Based on SDS-PAGE analysis summarized in Figure 1, PEF saliva has a wider variety of proteins than FEF, as revealed by the number of identified proteins (147 to PEF and 112 to FEF) as well as in number of spectral counts, which can represent a semi-quantitative approach (Table 1, 2 and 3). These data represent an apparent difference between PEF and FEF saliva. Interestingly, we observed high amounts of host proteins, which are presented predominantly in FEF saliva (Table 4). The tick proteins identified in this study were classified as (i) putative secreted proteins and (ii) putative housekeeping proteins, and were then divided into groups according to their molecular function (Tables 1, 2, 3 and Figure 2) consistent with previous published tick sialomes [4].

**Table 1:** Tick proteins identified in PEF saliva by *in solution* digestion.

Protein^a^ (75)	MW (kDa)	Spectral count	Coverage (%)	Best match BLAST^b^
**PUTATIVE SECRETED PROTEINS**				
**LIPOCALINS (9)**				
lipocalin 1	20.9	25	24	XP_002412631
lipocalin 2	21.0	19	20	ACX53907
lipocalin 3	20.4	14	13	XP_002412631
lipocalin 4	20.6	9	16	XP_002414294
lipocalin 5	14.8	9	21	DAA34565
lipocalin 35	24.7	7	11	ACX53907
lipocalin 36	22.8	5	10	ACX53955
lipocalin 46	26.4	4	10	ACX53986
lipocalin 6	20.3	3	9	ACX53907
**SECRETED CONSERVED PROTEINS (16)**				
secreted protein 27	37.5	51	29	XP_002403474
secreted protein 1	51.9	40	12	XP_002414081
secreted protein 39	15.5	39	32	ACX54027
secreted protein 28	30.6	29	35	AEE89467
secreted protein 2	13.5	27	22	XP_002424773
secreted protein 3	16.4	19	42	XP_002403368
secreted protein 4	23.2	17	14	XP_002435424
secreted protein 5	25.1	13	20	AAY66581
secreted protein 6	25.2	10	17	DAA34253
secreted protein 7	15.4	8	14	AEH03609
secreted protein 8	25.0	6	15	DAA34045
secreted protein 9	23.6	6	13	DAA34730
secreted protein 10	19.7	5	17	ACX53982
secreted protein 11	14.3	4	14	XP_002399909
secreted protein 12	26.2	4	8	XP_002414536
secreted protein 29	38.5	4	15	AEE89467
**PEPTIDASE INHIBITORS (3)**				
**Serpin**				
*R. microplus* serpin-6 (RmS-6)	44.4	52	31	XP_002402368
**Cystatin**				
cystatin 1	15.5	9	49	ACX53862
**Thyropin**				
thyropin 1	29.5	19	7	ACX54001
**ENZYMES (6)**				
**Peptidases**				
trypsin-like 1	39.5	9	6	XP_002435936
metallopeptidase 2	58.4	8	7	BAF43575
metallopeptidase 1	44.2	4	6	ADN23566
cathepsin B-like	38.5	2	8	BAF43801
**Phospholipases**				
phospholipase A2 1	44.8	17	10	XP_002399895
phospholipase A2 2	70.2	2	5	EFX77541
**GLYCINE-RICH SUPERFAMILY (12)**				
secreted cement protein 1	28.1	51	30	DAA34058
glycine-rich protein 1	13.9	16	36	AAV80791
cuticle protein 1	13.0	14	43	XP_002407787
glycine-rich protein 3	45.5	11	11	DAA34614
large GYY protein 3	15.0	7	27	XP_002411980
glycine-rich protein 4	9.2	7	25	XP_002411974
large GYY protein 1	14.0	5	22	XP_002411975
large GYY protein 2	15.8	3	20	XP_002411980
glycine-rich protein 5	9.1	3	35	XP_002411978
glycine-rich protein 2	32.0	3	8	DAA34246
proline-rich protein 1	66.7	2	3	XP_001942898
secreted cement protein 2	30.2	2	8	ACX54028
**ANTIGEN 5 PROTEIN FAMILY (1)**				
antigen 5/SCP domain-containing protein 1	45.9	33	21	XP_002403125
**TRANSPORTERS (1)**				
ferritin 1	21.4	10	21	ACJ70653
**CALRETICULIN (1)**				
calreticulin 1	47.8	5	11	AAR29940
**OTHER (1)**				
Kazal/SPARC domain-containing protein	32.7	13	21	XP_002413686
**PUTATIVE HOUSEKEEPING PROTEINS**				
**SIGNAL TRANSDUCTION (3)**				
metabotropic glutamate receptor 1	59.8	21	19	CAA67993
beta thymosin 1	6.9	5	30	ACX53929
inositol polyphosphate phosphatase	23.5	5	9	XP_002401241
**NUCLEAR REGULATION (2)**				
histone 2A 1	13.4	21	55	XP_002402622
RNA-binding protein	32.6	2	8	XP_002412054
**DETOXIFICATION (4)**				
Se-dependent glutathione peroxidase	17.7	63	67	AAY66814
peroxinectin 1	71.3	30	19	XP_002406316
glutathione S-transferase 1	25.6	7	12	AAD15991
glutathione S-transferase	16.8	2	17	AAQ74442
**CITOSKELETAL PROTEINS (4)**				
microtubule-associated protein 1	13.9	13	47	XP_002399901
tropomyosin 1	25.3	8	19	O97162
alpha tubulin 1	45.8	3	7	XP_002402152
actin-depolymerizing factor 1	17.0	3	17	AA34587
**PROTEIN SYNTHESIS. MODIFICATION AND EXPORT MACHINERY (5)**				
heat shock protein 70 1	54.3	4	11	DAA34064
heat shock protein 90 1	55.7	3	8	XP_002414808
40S ribosomal protein S28	11.6	3	21	ABR23349
14-3-3 protein zeta 1	28.1	3	10	Q2F637
heat shock protein 70 cognate	51.6	2	9	XP_002407132
**METABOLISM. NUCLEOTIDE AND CARBOHYDRATE (3)**				
alpha-L-fucosidase	50.5	10	14	XP_002412933
deoxyribonuclease II 1	45.1	4	14	XP_002399332
peptidyl-prolyl cis-trans isomerase 1	21.2	2	13	XP_002410624
**TRANSCRIPTION MACHINERY (1)**				
elongation fator-1 alpha 1	50.8	29	17	XP_002411147
**EXTRACELLULAR MATRIX AND ADHESION (3)**				
neural cell adhesion molecule 2	83.1	5	4	XP_002409358
fascilin-like protein	39.2	2	8	XP_002409988
beat protein-like 1	45.3	2	10	XP_002406531

a Accession numbers for tick identified proteins were deposited as Transcriptome Shotgun Assembly project at DDBJ/EMBL/GenBank under the accessions GBBO00000000 and GBBR00000000. The versions described in this paper are the first version, GBBO01000000 and GBBR01000000, respectively.

b Accession numbers of best matches identities obtained using BLASTP against the non-redundant protein database in GenBank.

**Table 2:** Tick proteins identified in FEF saliva by *in solution* digestion.

Protein^a^ (41)	MW (kDa)	Spectral count	Coverage (%)	Best match BLAST^b^
**PUTATIVE SECRETED PROTEINS**				
**LIPOCALINS (18)**				
lipocalin 49	16.0	56	59	ACX53907
lipocalin 37	16.7	35	41	XP_002414294
lipocalin 7	19.9	27	61	ACX53907
lipocalin 8	20.4	21	33	ACX53907
lipocalin 9	20.3	20	35	ACX53907
lipocalin 38	23.9	19	25	ACX53907
lipocalin 10	20.5	18	31	ACX53907
lipocalin 39	8.3	12	17	XP_002414617
lipocalin 40	24.9	11	18	ACX53986
lipocalin 47	16.3	10	28	ACX53907
lipocalin 41	15.5	8	29	ACX53986
lipocalin 11	20.3	8	23	ACX53907
lipocalin 12	19.9	6	13	ACX53907
lipocalin 13	19.9	5	12	XP_002406507
lipocalin 14	19.4	4	13	ACX53907
lipocalin 42	20.6	3	11	ACX53986
lipocalin 43	19.0	2	15	ACX53907
lipocalin 15	21.1	2	15	ACX53907
**SECRETED CONSERVED PROTEINS (8)**				
secreted protein 13	27.5	21	23	XP_002414536
secreted protein 14	13.2	20	33	XP_002413811
secreted protein 30	49.9	13	10	XP_002414081
secreted protein 40	6.6	10	11	BAG58161
secreted protein 16	6.9	8	23	YP_001186599
secreted protein 31	15.7	5	11	ACX54027
secreted protein 17	8.5	4	30	ZP_06826700
secreted protein 18	9.5	3	36	XP_001197477
**PEPTIDASE INHIBITORS (7)**				
**TIL domain-containing protein**				
TIL domain-containing protein 1	9.3	39	67	ACV83329
TIL domain-containing protein 2	9.2	17	53	ACV83329
TIL domain-containing protein 3	17.5	9	31	XP_002409984
TIL domain-containing protein 4	17.6	9	32	XP_002409984
**Thyropin**				
thyropin 2	28.2	4	10	ACX54001
thyropin 3	20.9	3	13	ACX54001
**Kunitz-type**				
Kunitz domain-containing protein 1	78.2	5	4	AAN10061
**IMMUNOGLOBULIN-BINDING PROTEIN (2)**				
immunoglobulin G-binding protein 2	19.9	18	26	XP_002414615
immunoglobulin G-binding protein 1	17.4	6	12	XP_002411824
**ENZYMES (2)**				
acetylcholinesterase 1	61.8	9	8	ADO65743
heme-binding aspartic peptidase (THAP)	40.5	5	6	AAG00993
**IXODEGRIN FAMILY (1)**				
cysteine-rich KGD motif-containing protein 1	19.0	5	5	XP_002411345
**CAP SUPERFAMILY (1)**				
cysteine-rich protein 2	17.5	35	31	XP_002411345
**IMMUNITY-RELATED PRODUCTS (1)**				
**Antimicrobial peptides**				
histidine-rich secreted protein 1	17.5	13	17	CAX82541
**TRANSPORTERS (1)**				
vitellogenin 1	201.8	13	6	AAA92143.1

a Accession numbers for tick identified proteins were deposited as Transcriptome Shotgun Assembly project at DDBJ/EMBL/GenBank under the accessions GBBO00000000 and GBBR00000000. The versions described in this paper are the first version, GBBO01000000 and GBBR01000000, respectively.

b Accession numbers of best matches identities obtained using BLASTP against the non-redundant protein database in GenBank.

**Table 3:** Tick proteins identified both in PEF and FEF saliva by *in solution* digestion.

			PEF		FEF	
Protein^a^ (70)	MW (kDa)	Spectral count	Coverage (%)	Spectral count	Coverage (%)	Best match NCBI^b^
**PUTATIVE SECRETED PROTEINS**						
**LIPOCALINS (23)**						
lipocalin 16	20.7	130	37	97	65	ACX53907
lipocalin 17	20.9	62	49	64	61	ACX53907
lipocalin 18	21.7	43	21	25	34	ACX53907
lipocalin 45	17.3	38	10	7	10	ACX53907
lipocalin 19	20.5	28	15	47	46	ACX53907
lipocalin 20	21.1	24	24	43	57	ACX53907
lipocalin 21	21.1	23	22	12	38	XP_002412631
lipocalin 22	20.5	23	17	33	27	XP_002415124
lipocalin 50	18.7	22	33	9	19	ACX53907
lipocalin 23	21.1	22	36	11	13	ACX53907
lipocalin 24	21.0	22	18	42	56	ACX53907
lipocalin 25	20.7	19	21	49	59	XP_002412631
lipocalin 48	15.4	18	30	5	18	ACX53907
lipocalin 26	20.6	17	27	43	45	ACX53907
lipocalin 27	19.8	16	18	33	28	ACX53907
lipocalin 44	23.9	14	5	25	25	ACX53907
lipocalin 28	20.7	11	16	8	10	XP_002414294
lipocalin 29	20.7	10	16	14	26	ACX53907
lipocalin 30	20.4	9	18	50	32	ACX53907
lipocalin 31	20.5	5	10	9	27	ACX53907
lipocalin 32	22.6	2	14	11	19	ACX53986
lipocalin 33	20.8	2	11	52	51	ACX53907
lipocalin 34	20.9	2	23	9	15	ACX53907
**SECRETED CONSERVED PROTEINS (15)**						
secreted protein 19	36.3	314	64	27	35	XP_002402717
secreted protein 20	25.1	82	44	48	42	DAA34225
Bm05	19.1	66	27	23	23	ABV53333
secreted protein 32	21.7	64	47	5	14	DAA34730
secreted protein 33	37.8	46	20	5	7	XP_002403474
secreted protein 21	72.2	45	59	19	47	XP_728368
secreted protein 22	15.8	44	18	16	13	ADN23561
secreted protein 34	37.5	37	21	15	16	XP_002402718
secreted protein 23	25.1	31	34	8	8	DAA34045
secreted protein 35	9.7	21	64	9	36	XP_002408964
secreted protein 36	21.6	16	12	14	16	XP_002414083
secreted protein 24	11.3	11	27	16	43	XP_002413811
secreted protein 25	16.7	7	16	10	21	XP_002408703
secreted protein 26	15.2	5	28	16	36	XP_002410662
secreted protein 37	42.4	2	7	9	13	XP_002411420
**PEPTIDASE INHIBITORS (10)**						
**Serpins**						
*R. microplus* serpin-17 (RmS-17)	43.2	206	77	37	32	ABS87360
*R. microplus* serpin-3 (RmS-3)	43.4	185	60	71	43	AAP75707
*R. microplus* serpin-3 (RmS-3)	43.4	175	65	66	39	AAK61377
*R. microplus* serpin-17 (RmS-17)	43.2	146	78	14	31	ABS87360
*R. microplus* serpin-6 (RmS-6)	44.3	68	41	28	18	ABI94056
*R. microplus* serpin-6 (RmS-6)	44.3	64	41	27	18	ABI94056
**Cystatin**						
RmCys2b	15.4	24	58	2	21	AGB35873
**Alpha2-macroglobulin**						
alpha2 macroglobulin 2	164.0	313	38	26	12	ACJ26770
alpha2 macroglobulin 1	85.1	20	11	8	4	XP_002405338
alpha2 macroglobulin 3	87.1	5	7	4	2	AAN10129
**ENZYMES (2)**						
chitinase 1	48.4	3	5	7	10	ACX33152
serine carboxypeptidase 1	35.6	2	6	7	7	XP_002404034
**8.9 kDa FAMILY (2)**						
8.9 kDa protein 1	11.7	24	12	12	19	ACG76246.1
8.9 kDa protein 2	11.7	13	8	7	36	ACX53877
**MUCIN (1)**						
mucin 1	25.5	47	26	5	7	AAA97877
**IMMUNITY RELATED PRODUCTS (4)**						
**Antimicrobial peptides**						
microplusin-like 2	10.7	125	40	34	45	AAY66495
BmSEI-like 1	11.5	51	51	167	63	ABH10604
BmSEI-like 2	11.0	29	21	185	43	ABH10604
microplusin-like 1	16.0	8	9	4	19	ABB79785
**TRANSPORTERS (8)**						
hemelipoprotein HeLP	146.8	3945	77	353	47	ABK40086
hemelipoprotein HeLP 3	94.0	2810	72	207	39	ABK40086
vitellogenin 2	19.0	601	44	6	4	XP_002401768
hemelipoprotein HeLP 2	30.6	512	61	42	47	ABK40086
vitellogenin 4	10.8	381	48	32	16	BAJ21514
vitellogenin 5	64.4	77	36	2	4	XP_002401765
salivary lipid-interacting protein 1	20.4	61	27	7	21	XP_002414779
vitellogenin 3	21.7	9	4	28	10	BAH02666
**PUTATIVE HOUSEKEEPING PROTEINS**						
**CYTOSKELETAL PROTEINS (1)**						
actin 1	41.8	99	39	15	22	AAP79880
**IMMUNITY RELATED PRODUCTS (1)**						
Toll-like receptor 5	38.3	22	12	2	6	DAA34254
**EXTRACELLULAR MATRIX AND ADHESION (3)**						
ML domain-containing protein 1	13.5	28	35	10	18	XP_002434499
neural cell adhesion molecule 3	40.5	13	26	5	16	XP_002414299
neural cell adhesion molecule 1	63.1	16	8	3	4	XP_002409358

a Accession numbers for tick identified proteins were deposited as Transcriptome Shotgun Assembly project at DDBJ/EMBL/GenBank under the accessions GBBO00000000 and GBBR00000000. The versions described in this paper are the first version, GBBO01000000 and GBBR01000000, respectively.

b Accession numbers of best matches identities obtained using BLASTP against the non-redundant protein database in GenBank.

**Table 4:** Host proteins identified in PEF and FEF saliva by *in solution* digestion.

		PEF		FEF	
Protein (68)	Accession number	Spectral count	Coverage (%)	Spectral count	Coverage (%)
**PEF (17)**					
actin, alpha skeletal muscle	IPI00697648.1	64	24	-	-
allergen Bos d 2	IPI00708946.1	38	36	-	-
keratin, type I cytoskeletal 14	IPI00721270.4	36	14	-	-
beta actin	IPI00905257.2	34	17	-	-
keratin, type II cytoskeletal 75	IPI00700471.2	26	9	-	-
keratin, type II cytoskeletal 7	IPI00694214.1	20	7	-	-
odorant binding protein-like	IPI00722909.1	20	41	-	-
histone H2A	IPI00698058.5	16	35	-	-
keratin, type II cytoskeletal 79	IPI00707469.2	16	4	-	-
keratin 15	IPI00692588.3	15	6	-	-
KRT4 protein	IPI00709590.5	13	7	-	-
secretoglobin	IPI00838546.1	10	26	-	-
keratin, type I cytoskeletal 24	IPI00698285.3	8	6	-	-
histone H4 replacement-like	IPI00716205.3	8	17	-	-
heat shock protein HSP 90-alpha	IPI00699622.3	5	5	-	-
lipocalin 2 (oncogene 24p3)-like	IPI00685784.3	2	14	-	-
annexin A1	IPI00703345.2	2	8	-	-
**FEF (38)**					
serotransferrin	IPI00690534.1	-	-	87	38
alpha-2-macroglobulin	IPI00871133.1	-	-	54	19
immunoglobulin kappa light chain	IPI00699011.3	-	-	46	42
immunoglobulin light chain	IPI01028259.1	-	-	41	42
immunoglobulin light chain	IPI00838162.2	-	-	40	44
immunoglobulin light chain	IPI00855695.1	-	-	40	39
immunoglobulin light chain	IPI00867205.1	-	-	40	44
fibrinogen gamma chain	IPI00843209.1	-	-	33	33
immunoglobulin M heavy chain	IPI00714264.4	-	-	29	27
fibrinogen beta chain	IPI00709763.5	-	-	28	22
fibrinogen alpha chain	IPI00691819.1	-	-	25	15
complement C3 (Fragment)	IPI00713505.2	-	-	24	10
SERPINA1 Alpha-1-antiproteinase	IPI00695489.1	-	-	20	17
SERPINA3-2 Serpin A3-2	IPI00930024.1	-	-	20	19
apolipoprotein A-I	IPI00715548.1	-	-	17	35
immunoglobulin iota chain-like, partial	IPI00907960.2	-	-	17	24
carbonic anhydrase 2	IPI00716246.2	-	-	15	22
SERPINA3-1 Uncharacterized protein	IPI00968658.1	-	-	15	23
SERPINA3-3 Serpin A3-4	IPI00971592.1	-	-	15	17
serpin A3-7 isoform X1	IPI00971595.1	-	-	15	19
SERPINA3 Serpin A3–5	IPI00707034.6	-	-	14	16
hemopexin	IPI00690198.4	-	-	12	14
SERPINA3-6 Serpin A3-6	IPI00829575.1	-	-	12	15
immunoglobulin lambda-like polypeptide 1-like	IPI01002118.1	-	-	11	15
immunoglobulin light chain	IPI00718725.5	-	-	10	19
SERPINA3-7 Endopin 2C	IPI00705594.1	-	-	9	11
peroxiredoxin-2	IPI00713112.1	-	-	9	23
transthyretin	IPI00689362.1	-	-	8	35
cathelicidin-2	IPI00691669.1	-	-	6	16
alpha-2-HS-glycoprotein	IPI00707101.1	-	-	6	13
protein unc-45 homolog A	IPI00716476.2	-	-	6	3
vitamin D-binding protein	IPI00823795.1	-	-	5	11
cathelicidin-4	IPI00686754.1	-	-	4	19
immunoglobulin kappa light chain	IPI00889485.1	-	-	4	19
zinc finger CCCH domain-containing protein 7B	IPI00693044.4	-	-	3	1
flavin reductase	IPI00718510.2	-	-	3	13
immunoglobulin kappa light chain	IPI00906505.1	-	-	3	19
AF4/FMR2 family member 3	IPI01017768.1	-	-	2	3
**PEF and FEF (13)**					
hemoglobin subunit beta	IPI00716455.1	210	66	258	74
serum albumin	IPI01028455.1	174	31	452	64
serum albumin	IPI00708398.2	164	30	431	64
hemoglobin subunit alpha	IPI00710783.2	152	77	230	90
keratin 6A-like	IPI01002591.1	44	8	20	10
KRT6A protein	IPI00845184.1	41	12	7	4
keratin 13-like isoform 2	IPI00912554.1	36	14	32	10
keratin 6A-like	IPI01001566.1	29	5	9	3
keratin 2-like	IPI01003176.2	28	5	10	5
keratin, type II cytoskeletal 5	IPI00697851.1	18	4	7	4
polyubiquitin-C	IPI00726431.1	11	4	12	4
cathelicidin-1	IPI00718108.1	9	23	8	30
peptidoglycan recognition protein 1	IPI00701640.1	7	13	6	30

### Hemelipoprotein and other transporter proteins

Hemelipoproteins are the most abundant proteins in PEF and FEF saliva, based on protein band intensity (Figure 1) and spectral count (Table 3). In SDS-PAGE, these proteins appeared as two predominant bands between 95 and 130 kDa (Figure 1) consistent with a previous study that reported that the major hemelipoprotein present in *R. microplus* hemolymph (HeLp) consists of two subunits (92 and 103 kDa) [46], [47]. Although HeLp has no full-sequence deposited in any protein database, peptides corresponding to N-terminal sequence of HeLp subunits match the sequences for hemelipoproteins identified in tick saliva here, corresponding to HeLp-A and HeLp-B subunits [46]. HeLp has the ability to bind eight heme molecules, the prosthetic group released from hemoglobin digestion, and deliver them to tick tissues [46]. As a predominant protein in hemolymph, the presence of HeLp in *R. microplus* saliva could be explained by the phenomenon of hemolymph components incorporation by salivary glands, leading to secretion in saliva [48]. However, in other tick species, the transcriptional profile and protein localization of these hemelipoproteins in salivary glands of adult and unfed ticks suggest that they could act in different pathways during blood-feeding [18], [49], [50]. Previous studies have described these proteins in saliva from other ticks, which indicates that they are a conserved feature among different tick species [17], [18], [20], suggesting that HeLp may play vital role(s) in tick feeding and survival.

Since this protein could transport other compounds such as cholesterol, phospholipids and free fatty acids, in addition to heme [47], it is possible that they are secreted in the feeding site carrying small pharmacologic active molecules. It may also be postulated that hemelipoproteins perform non-classical yet unknown functions at the tick-feeding site. Recently, the main hemelipoprotein form in *Dermacentor marginatum* was shown to be a carbohydrate-binding protein with galactose- and mannose-biding specificity able to agglutinate red blood cells [51]. In addition, as ticks use the pool-feeding strategy to feed [39], hemolysis at the feeding site is plausible due to the presence of digestive peptidases in saliva (Table 1 and 2). It is known that both heme and the heme-binding protein hemopexin have pro-inflammatory and anti-inflammatory properties, respectively [52]–[54]. Thus, the presence of hemelipoproteins could lower free heme concentration at the feeding site, preventing inflammation.

It may be speculated that HeLp is also essential to heme storage and/or detoxification in ticks. An important adaptation that co-evolved with blood feeding is heme sequestration by heme-binding proteins and heme excretion, both of which prevent oxidative stress and tissue damage [55]. Interestingly, *R. microplus* ticks are unable to synthesize heme *de novo* [56], so hemelipoproteins could be critical components of a mechanism for sequestration, storage and utilization of host heme [46], [49]. Due to their high concentration in tick saliva, it is possible that relatively high concentrations of hemelipoproteins are present at the feeding site. This may allow re-ingestion of these proteins along with blood. In this scenario, hemelipoproteins may act as heme transporter when hemoglobin digestion begins in the midgut, since the high content of heme in the cytosol of midgut cells suggests a heme transport pathway from the digestive vesicles through the cytosol to reach the midgut basal surface, where heme is transferred to hemolymph to be delivered to the ovary [57], [58]. These molecules may be internalized in midgut cells by endocytosis, mediated by specific receptors, as described in mammal cells (e.g. heme-carrier protein hemopexin) [59]. This hypothesis is supported by the results of midgut proteome analysis of *Dermacentor variabilis*, where a hemelipoprotein was identified by LC-MS/MS, but not in the midgut cDNA library [60], suggesting that this protein is delivered from other tissue/secretion. Furthermore, *D. marginatus* major hemolymphatic hemelipoprotein was immuno-localized inside the midgut cells [51]. In the same way, hemelipoproteins may act in an excretory system to remove heme excess, obtained from blood ingestion, binding heme and re-injecting it into the host. This hypothesis of heme-binding agrees with the fact we detected a high amount of hemelipoproteins in PEF than in FEF saliva, and this reduction of hemelipoproteins in FEF saliva was accompanied by an increase in the host heme-binding proteins (Figure 1, Figure 2, Table 3 and Table 4). These findings are compatible with a mechanism in which, towards the end of feeding, the tick replaces hemelipoprotein as heme-carrier by host derived heme-carrier proteins, including serum albumin, hemopexin, apolipoprotein and peroxiredoxin (Figure 2 and Table 4). This may be possible at this stage because, after completing feeding, hemelipoproteins are necessary for vitellogenesis [61]. However, the presence of heme in tick saliva is yet to be demonstrated and needs further investigation. Similarly, ferritin is present only in PEF saliva (Table 1). Ferritin is an important iron reservoir, working as a protective mechanism against free iron overload. It is considered to be crucial for *Ixodes ricinus* development and reproduction [62], [63]. Apparently, the absence of ferritin in FEF saliva is functionally compensated by serotransferrin, an iron-carrier protein from the host (Table 4). These observations strongly suggest the existence of a cooperative system between tick and host carrier-proteins, especially those involved in heme and/or iron regulation during blood-feeding. The role of these proteins in tick-host needs further investigation

### Lipocalins

Lipocalins are single modular proteins of around 200 amino acids that fold tightly in a β-barrel with potential for binding small hydrophobic molecules in a central pocket. The tertiary structures of lipocalin are greatly conserved, even when amino acid sequence similarities are low [64], [65]. In most organisms lipocalins are characterized by the consensus structural conserved regions (SCRs) that are characteristic of kernel lipocalins [66], while tick proteins assigned to the lipocalin family lack the typical SCR [67]. Annotation of the most recently identified tick lipocalins is based on homology with annotated histamine-binding proteins from other tick species, based on the presence of the characteristic tick histamine-binding domain (PF02098) as described in the Pfam database [37], [67]–[69]. PEF and FEF *R. microplus* secrete 50 different lipocalins in saliva (Table 1, 2 and 3). From these identified lipocalins, except for lipocalin 5, which matches the lipocalin domain (PF00061), all other identified *R. microplus* lipocalins possess the tick histamine-binding domain (PF02098), when scanned against the Pfam database or when visually inspected (data not shown) [37], [69], [70]. MS/MS data show that saliva lipocalins spectral counts are higher in FEF than in PEF (Table 1, 2 and 3). The presence of high amounts of lipocalins in cattle tick saliva is comparable with data from the *O. moubata* saliva proteome, showing that lipocalins are the most abundant salivary protein in this species [17]. Some of these *R. microplus* identified lipocalins have similarities with some described tick lipocalins, which have antihemostatic and immunomodulatory activities [68], [69], [71]–[80], such as amine-binding molecules. The high content of lipocalins in tick saliva is compatible with their antihemostatic and immunomodulatory roles during tick parasitism [4]–[7]. Since histamine and serotonin secreted by the host at the feeding site induce cutaneous inflammation, ticks have to overcome their activities in order to complete feeding [4]–[7]. Sequestering these host molecules may be a mechanism used by *R. microplus* against these defensive reactions that affect thick attachment to hosts [81], [82]. The high content of lipocalins in *R. microplus* saliva also could be related to level necessary to block the near micromolar concentration of biogenic amines and prostaglandins that accumulate at the feeding site [4]. The importance of this mechanism for tick feeding is underlined by the fact that *R. microplus*-resistant cattle have its status reverted to susceptible when treated with anti-histamines (H1 antagonists) [83]. Besides, a recent study that demonstrated that tick-resistant cattle sera have a higher IgG titer against lipocalins, compared to susceptible animals, stresses the importance of this class of proteins for blood-feeders [70]. The presence of a high concentration of lipocalins in FEF (Table 1, 2, 3 and Figure 2) is intriguing, because at this stage blood sucking is completed, and the tick does not need to modulate host defense mechanisms. It is possible that lipocalins found in FEF saliva signal the role(s) of these molecules during the last stages of the rapid feeding phase, when the tick takes huge amounts of blood or prepares to detach from host skin.

### Secreted conserved proteins

Transcriptomical analyses of salivary gland of hard and soft ticks have provided reliable data on blood-feeding behavior [4], [5], [7]–[16]. The repertoire of tick salivary gland transcripts found is much broader and complex than anticipated, with many proteins without similarities to proteins in the NCBI database. Most of these new proteins were identified just as hypothetical secreted conserved proteins [4]. Proteins included in this group are the most abundant proteins in *R. microplus* saliva, and PEF saliva is richer in these proteins than FEF saliva (Table 1, 2, 3). The presence of these proteins in *R. microplus* saliva, as observed in the present study, confirms that some previously described hypothetical secreted conserved proteins are actually secreted proteins. Members of this type of proteins in *R. microplus* are 70–460 amino acid proteins (predicted molecular weight varying from 6.6 to 51.9 kDa) and some of them migrate as 34–60 kDa proteins when separated in SDS-PAGE (Figure 1 and Table S1), suggesting that they have post-translational modifications. Given the higher number of these proteins present in tick saliva, it is reasonable to conclude that they have a role in tick feeding. The *A. americanum* AV422 protein (*Aam*AV422) is a member of the secreted conserved protein group that is differentially up-regulated in response to contact with host and/or exposure to feeding stimuli [84], [85]. This protein is secreted and injected in the host within the first 24 h of tick attachment onto the host. Apparently, *Aam*AV422 is involved in the mediation of tick anti-hemostasis and anti-complement functions, since r*Aam*AV422 delays plasma clotting time in a dose responsive manner, prevents platelet aggregation and reduces the formation of terminal complement complexes [84], [85]. *R. microplus* secreted protein 20 is 99% identical to *Aam*AV422, and is secreted in PEF and FEF saliva (Table 3). Like *Aam*AV422, it may act as an anti-hemostatic and anti-complement protein [85]. Further studies are necessary to better characterize this group of salivary proteins, and may represent an opportunity to discover new targets for parasite control.

### Peptidase inhibitors

The tick feeding style of lacerating host tissue and sucking host blood from the pool formed at the bite site is expected to strongly trigger host defense responses as hemostasis, inflammation, and complement systems [4], [5], [86]. These responses are dependent on the action of several peptidases, such as procoagulant (thrombin, factor Xa and other coagulation factors), pro-inflammatory (neutrophil elastase, proteinase-3, chymase, tryptase, kallikrein, cathepsin L, cathepsin B, cathespin S, cathepsin C and cathepsin G) and complement enzymes (factors B, C, D and component 2) [4], [5], [86], [87]. These host defenses are highly regulated by specific endogenous inhibitors, maintaining homeostasis. From this perspective, it has been suggested that ticks secrete peptidase inhibitors to disrupt host defenses, facilitating feeding [88].

#### Serpins

proteins that belong to the serpin (serine protease inhibitor) superfamily are expressed in all branches of life [89]. They have a role in the control of several endopeptidase cascades in many organisms [90]. In mammalians, most serpins play crucial roles, controlling endopeptidases involved in blood coagulation, fibrinolysis, inflammation, and complement activation [89], [91]. It is assumed that tick secreted serpins disrupt host homeostatic balance in order to facilitate parasitism [88]. Recently, 18 full-length serpin encoding sequences were described in *R. microplus* [92], three of which (RmS-3, RmS-6 and RmS-17) were identified in PEF and FEF saliva (Table 3). Notably, PEF saliva has a high number of spectral counts of this protein family (Table 3), suggesting that inhibition of serine endopeptidases involved in host defense system is important earlier in blood tick feeding. It was shown that tick-resistant cattle sera have high titers of antibodies against RmS-3, compared to tick-susceptible animals, suggesting its importance in the tick-host relationship [93]. Furthermore, the administration of an antibody against RmS-3 linear epitope by artificial feeding decreases the reproductive capacity of *R. microplus* females by 81% [93]. However, the precise role of these inhibitors in *R. microplus* saliva remains unclear. The presence of these serpins in *R. microplus* saliva could be responsible, at least partially, for the anti-thrombin [94] and anti-thrombotic [95] properties of its saliva, including their local and systemic alterations [26]. Moreover, some other pharmacological activities of *R. microplus* saliva may be associated to serpins, such as immunomodulatory activity [96]–[99]. The potential effect of these proteins on host systems are supported by several studies showing serpins from hematophagous parasites act as anti-coagulant and anti-inflammatory agents, being essential for a successful blood meal [96]–[102]. Clearly, data showing that the use of serpins as vaccinal antigens impairs tick development reinforces the importance of these proteins in regulating tick physiology [103]–[107].

#### α2-macroglobulin (α2M)

these are large glycoproteins and are present in the body fluids of both invertebrates and vertebrates, being secreted as glycosylated polypeptides with a molecular mass of about 180 kDa [108]. Three α2M were identified in PEF and FEF saliva (Table 3), and based on spectral counts all three seem to be most abundant in PEF, relatively to FEF. In vertebrates, α2M proteins have been found to regulate host cell apoptosis [109], inhibit several serum peptidases like thrombin [110], factor Xa [111] and kallikreins [112], mediate T-cell proliferation [113] and induce proliferation and activation of macrophages [114]. Tick saliva α2M may be linked to interference in inflammation and immunomodulation, and it may be an additional salivary anti-coagulant. It is still unclear whether these α2M act as immunomodulators or as anticoagulants, this role needs to be elucidated. However, the fact that such inhibitors (as α2M proteins and serpins) are secreted mostly in PEF saliva (Table 3) reinforces the idea that inhibition of host-defenses endopeptidases is important as early as in the beginning of the blood meal.

#### TIL domain-containing proteins

proteins belonging to the TIL (trypsin inhibitor-like) domain-containing group have been reported in blood-feeding mosquitoes and tick sialomes [5]. Ixodidin, an example of this group of inhibitors, was isolated from *R. microplus* hemolymph. In addition to antimicrobial activity, ixodidin has anti-trypsin and anti-elastase activities [115]. Only FEF saliva has peptides matching this group of proteins, including ixodidin (Table 2). These proteins may act similarly to host endopeptidases inhibitors, increasing the inhibition of the target endopeptidases. Additionally, presence of these proteins at the final phase of blood meal acquisition suggests that they have a possible role as an antimicrobial protein to prevent (or control) infection in ticks after blood-meal acquisition. Their interfering role in tick-vectoring ability, regulating the quantity or even the specificity of pathogens ticks transmit remains to be addressed.

#### Thyropin

thyropin (thyroglobulin type-1 domain protease inhibitors) is a family of proteins characterized by the presence of thyroglobulin type-1 domain repeats [116], [117]. The well characterized type-1 domain-containing protein was described in the sea anemone *Actinia equina* and has been shown to inhibit either cysteine or cation-dependent peptidases [118], including cathepsin L, cathepsin S, papain and cruzipain [117], [119]. PEF and FEF saliva contains three thyropins (Table 1 and 2). It is possible that these proteins inhibit some host cysteine endopeptidases, contributing to the immunomodulatory effects of tick saliva. This hypothesis has yet to be proved, since thyropins have not been functionally characterized in ticks to date. Proteins containing these domains are present in several tick sialomes [4], and their presence was previously also detected in *O. moubata* and *R. sanguineus* saliva [17], [19].

#### Cystatin

cystatins comprise a large family of reversible and tight-binding inhibitors of papain-like enzymes and legumains [120], which are involved in biological processes like antigen processing and presentation, phagocytosis, neutrophil chemotaxis during inflammation and apoptosis [121]–[124]. Two proteins of the cystatin family were identified in PEF and FEF, with higher spectral counts in PEF saliva (Table 1 and 3). The most abundant (RmCys2b – AGW80658.1) is a member of type 2 cystatin [125] and is present predominantly in PEF saliva (Table 3). It is able to inhibit cathepsin B, cathepsin L and cathepsin C (L. F. Parizi, personal communication). As these enzymes are important in some immunologic processes, these cystatins in *R. microplus* saliva could act as immunomodulators during the slow feeding phase of cattle tick parasitism, as previously shown for other tick cystatins, facilitating blood feeding and pathogen transmission [126]–[130]. The importance of these inhibitors in blood feeding was underscored in studies that showed that neutralization of cystatins (through gene silencing in ticks or vaccines) significantly reduces tick feeding ability [128], [131], [132].

#### Kunitz-type inhibitors

members of the Kunitz-type family are particularly well characterized as inhibitors of a large number of serine endopeptidases [133]. One protein containing Kunitz domains was found only in FEF saliva (Table 2). Interestingly, this protein contains nine *in tandem* Kunitz domains, a remarkable difference among well characterized inhibitors of this class in other ticks, which range between one and five domains [25], [134]. These inhibitors have been characterized as acting upon thrombin, factor Xa, factor XIIa, trypsin and elastase [25]. This raises the suggestion they contribute to *R. microplus* saliva anticoagulant activity [26], [94], [95].

### Glycine-rich proteins

This group of proteins is described in several tick sialomes and has distinct subdivisions [4]. In ticks, proteins containing glycine-rich (Gly-rich) and proline-rich (Pro-rich) repeat motifs are associated with tick-cement functions [135], [136]. Ten proteins of this superfamily were found exclusively secreted in PEF saliva (Table 1). These proteins have been identified also in *O. moubata* and *R. sanguineus* saliva [17], [19]. The presence of these proteins at this stage lends strength to the hypothesis that they are important in the formation of a cement cone that affords tick attachment to the host during initial feeding phase. Three of these proteins contain the motif [LPAE]-P-G, that are known as targets of proline hydroxylase (data not shown) [137], [138], a post-translational modification which allows cross-linking between proteins, a characteristic present in cement proteins [139]. The identification of these proteins at this developmental stage is in accordance with a previous study on *A. americanum*, where genes codifying for this superfamily of proteins are up regulated at the early stages of parasitism [84].

### Enzymes

#### Peptidases

parasite secreted enzymes may play a wide array of roles in host tissues. Analysis of PEF tick saliva allowed the identification of two metallopeptidases (Table 1). In this sense, metallopeptidases, frequently associated with vascular damage, tissue remodeling and degradation of serum compounds [140] may have a role modulating host responses against ticks. As shown in other ticks, this salivary metallopeptidases may be linked to fibrin(ogen)lysis [141], bradykinin degradation [142], and angiogenesis inhibition [143]. In PEF saliva, a trypsin-like enzyme similar to factor-D from *D. variabilis* was identified (Table 1). This enzyme may interfere with host inflammation and blood clotting, acting as plasminogen activator or protein C activator, similarly to what has been reported for *I. scapularis* saliva [144]. The secretion of metallopeptidases and trypsin-like enzymes in tick saliva is stage-dependent, since the analysis performed here indicates that FEF saliva does not have significant amounts of these enzymes. The presence of these proteins in PEF saliva could also be explained by the fact that host defense modulation is crucial for blood feeding at this time.

In FEF tick saliva, only one endopeptidase was identified, the tick heme-binding aspartic peptidase (THAP) (Table 2). Here, we report, for the first time, the presence of THAP in cattle tick saliva. THAP is able to hydrolyze hemoglobin and vittelin, and thus is supposed to have a role in *R. microplus* digestion and embryogenesis [145], [146]. It may be hypothesized that THAP acts as a digestive enzyme secreted in the host during the fast engorgement phase. During blood meal acquisition, THAP may start the digestion process of blood components in the hemorrhagic pool at the tick attachment site. Similarly, this activity could explain the presence of a cathepsin-B in PEF saliva (Table 1), as this type of enzymes has been described to hydrolyze hemoglobin in other tick species [147], [148]. In the same way, saliva of both PEF and FEF secretes a serine-carboxipeptidase (Table 3). Since a serine-carboxipeptidase from midgut was able to hydrolyze bovine hemoglobin in *Haemaphysalis longicornis*, it suggests that it also may be involved in digestion of the blood meal at feeding site [149]. In this way, the presence of these digestive enzymes in saliva may be associated with the presence of heme-binding proteins, since the free-heme delivered by hemoglobin digestion at the feeding site has to be sequestered, because heme has pro-inflammatory properties [52] and impairs blood meal acquisition.

#### Phospholipase A2

phospholipases A2 (PLA2) are secreted enzymes that have been implicated in several biological processes, such as modification of eicosanoid generation, inflammation and host defense [150], [151]. Two PLA2 proteins were found in PEF saliva (Table 1). Secretory PLA2 are common and important components of bee and snake venoms, and have hemolytic, antiplatelet aggregation, and anticoagulant effects through their ability to interact with cells or by the degradation of phospholipid, thus generating free arachidonic acid [152]. Likewise, in *A. americanum* these proteins are suggested to act in the hemolytic activity of saliva [153], [154]. The presence of PLA2 in PEF is in accordance with those digestive enzymes described above, which also may play a role in host blood cells lyses, facilitating the tick digestive process at feeding site. Additionally, these enzymes may act as antiplatelet and anticoagulant agents [152], facilitating blood feeding and reinforcing the notion that defense modulation in PEF is crucial for blood feeding.

### Immunity-related proteins

#### Antimicrobial peptides

antimicrobial peptides (AMPs) are widely distributed in nature and are essential components of the first defense line against infections [155]. In invertebrates, which have only innate immunity, AMPs are extremely effective and work as powerful weapons against bacteria and fungi [156]. Microplusin is an AMP from *R. microplus* that belongs to the group of cysteine-rich AMPs with histidine-rich regions at N- and C-termini, which have been implicated in sequestration of zinc, a microbial growth factor [157], [158]. Proteins of the microplusin-like and histidine-rich families are present in the saliva of both PEF and FEF (Table 2 and Table 3). The role(s) of these proteins in tick saliva may be associated with the prevention of microbial proliferation at the tick-feeding site. Moreover, since a lot of saliva is ingested together with the diet, especially in pool feeders, it could be assumed that the AMP may also act in the midgut of ticks.

### Putative housekeeping proteins

In *R. microplus*, we identified putative housekeeping proteins, predominantly in PEF saliva (Table 1 and 3). Putative housekeeping proteins in tick saliva have been identified in *O. moubata* and *R. sanguineus* [17], [19]. The presence of this kind of protein in tick saliva is supported by observations showing apocrine and merocrine secretion in tick salivary glands [159]. Moreover, these housekeeping proteins can be secreted in non-classical pathways to the extracellular environment [160], [161]. Presence of these proteins in tick saliva is underlined by the fact that hosts infested with *A. americanum* develop antibodies against housekeeping proteins during different tick feeding stages (A. Mulenga, personal communication).

The presence of housekeeping proteins in tick saliva may have further biological importance, since these proteins may play different roles in the tick-host interface. For example, since HSP70 is present in PEF saliva, it may be involved in tick-host relationship (Table 1). In an experimental model of disease, HSP70 administration prevents inflammatory damage and promotes the production of anti-inflammatory cytokines [162]. Similarly, a study showed that HSP70 from *Mycobacterium turbeculosis* has anti-inflammatory properties, inhibiting pro-inflammatory cytokine production by IL-10 driven down-regulation of transcriptional factor in dendritic cells [163]. Other examples of housekeeping protein involve enzymes linked to detoxification (Table 1). Glutathione S-transferase (GST) is a protein that catalyzes the conjugation of glutathione with several xenobiotic and endogenous substances [164]. In this sense, GST seems to be closely associated with detoxification and acaricide resistance [165]. Additionally, it has been proposed that GST secreted by parasite salivary glands has immunomodulatory activity due to the alteration of cytokine gene expression profile, modulation of immune cell proliferation and decrease in oxidative ability of phagocytes [166]. Further studies are necessary to elucidate the role of this class of proteins in tick saliva, since this appears to be a conserved feature among different tick species [17], [19].

### Host proteins

A large number of bovine proteins were identified in the saliva of both PEF and FEF, being present predominantly in FEF saliva, relatively to PEF saliva (Table 4). The presence of host proteins in tick saliva has been reported in other ticks species [17]–[20]. These proteins are the majority secreted proteins in *R. sanguineus* saliva [19]. It was demonstrated that ticks transport intact proteins across the digestive system to the hemolymph [167]. Furthermore, some of the host proteins described in *R. microplus* proteome have been found in salivary glands of other tick species [12], [18], [20], [48], suggesting that the presence of host proteins in tick saliva may be a real and common recycling system present in ticks, not a result of contamination during saliva collection. Furthermore, the presence of different classes of host proteins in the saliva of the two tick developmental stages suggested the existence of this selective uptake process (Table 4 and Figure 2). For example, in PEF saliva we observed a predominance of housekeeping proteins (actin, nuclear proteins like histone and HSP90) and hemoglobin subunits peptides (Table 4 and Figure 2). In FEF saliva this pattern switches dramatically due to: (i) transporter and/or proteins associated with metabolism of heme and iron, like serum albumin, peroxiredoxin, serotransferrin, apolipoprotein and hemopexin; (ii) immunity, like immunoglobulins chains and C3 complement protein; (iii) peptidase inhibitors of the serpin superfamily; and (iv) other proteins (Table 4 and Figure 2). Similarly, rabbit proteins involved in heme and iron metabolism (as serum albumin, serotransferrin and hemopexin); immunity (C3 complement protein); and serpins were identified in *R. sanguineus* saliva [19]. However, as in *R. sanguineus* saliva was collected from 5–7 days partially fed adults ticks [19], it is not possible to compare these differences among different developmental stages, as found in *R. microplus*.

We are mindful of the possibility that tick saliva proteins in FEF may not represent exactly what occurs at the end of the blood feeding. However, it is remarkable that the majority of host proteins in FEF saliva have heme-binding and endopeptidase inhibitory functions similar to some of the tick proteins in PEF saliva (Figure 2). A quite interesting question is: if these proteins are returned intact, can they exert their biological function in the host? For instance, mammalian serpins were detected in FEF saliva (Table 4), so the question is: do these host serpins inhibit host serine endopeptidases of defense pathways as the tick prepares to detach? Whether these proteins are returned to the host as intact proteins or products of partial hydrolysis remains to be clarified. However, as in *R. sanguineus* saliva [19], it seems that host serum albumin is secreted intact into the host, since SDS-PAGE analysis reveals a ∼60 kDa protein (Figure 1), which is intact [168]. Taken together with previous results that show the existence of a separate pathway for uptake and digestion of albumin in relation to hemoglobin incorporation into midgut cells [57], these results may be evidence of the existence of a system to recycle serum albumin. However, if serum albumin secreted into host is carrying some molecule along needs to be further clarified. In addition, it is important to note that several of these mammalian proteins, when undergoing limited proteolysis, generate peptides, some of which are bioactive, presenting antimicrobial action [169], [170], as well as vasoactive peptides [171] which may enhance parasitism.

The presence of immunoglobulin chains in tick saliva could be explained as a part of the tick self-defense system, since immunoglobulin remains as an active protein in tick hemolymph [172]. In addition, the existence of immunoglobulin-binding proteins in both the tick salivary gland and hemolymph indicates that hemolymph and salivary gland cooperate to remove foreign proteins that could be deleterious for tick development during feeding [48]. An observation that support this hypothesis is that, in *R. microplus*, immunoglobulin-binding proteins from tick were found in the same developmental stage at which host immunoglobulin was found, in FEF saliva (Table 2 and 4). Differently from *R. microplus*, saliva immunoglobulin was not identified in *R. sanguineus* [19]. In spite of that, as these proteins were identified only in FEF in *R. microplus*, the presence in FEF saliva of *R. sanguineus* cannot be ruled out.

Despite reports of the presence of host proteins in tick saliva, this remains a neglected issue in the study of tick biology. It is interesting to note that while long-term blood feeders like *R. microplus* and *R. sanguineus* saliva contains considerable amounts of host proteins, the saliva of the short-term blood feeder, such as *O. moubata*, contains only a few host proteins [17], [19]. The demonstration of these proteins in tick saliva raises several questions to be further explored, and may reveal novel insights into tick-host relationship.

## Conclusion

The advancements in transcriptomic and proteomic analyses in recent years have opened unprecedented opportunities to identify putative targets for tick control into the variety of tick salivary transcripts and proteins. Saliva of ticks are far more complex than anticipated, having hundreds of different tick proteins as well as a high content of host proteins, which could have a role in several pathways associated with tick survival. A complete identification of tick salivary compounds and their identification and characterization remains a major research challenge that will help understand how host modulation by ticks occurs. The proteomic approach allows a comprehensive analysis of saliva composition and provides novel information to guide further studies about molecular, biochemical, immune biological, pharmacological as well as physiological characterization of these proteins. In *R. microplus* it is technically challenging to study defined feeding time points, and this is the reason why all previous studies have utilized saliva of fully engorged ticks. It is conceivable that after detaching from the host (or most probably just before detaching) ticks stop secreting proteins, indeed, salivary gland degeneration starts at this point. So, all studies conducted with saliva or salivary glands from FEF ticks must be carefully interpreted. This study, comparing saliva from PEF and FEF ticks, helps identify tick proteins that are important in the tick feeding process. These data could contribute to the understanding of tick salivary gland physiology and the tick-host relationship as well clues to approach new immunologically based tick control.

To date, only a few reports have explored *R. microplus* saliva. Compared to other hematophagous parasites, there is relatively little information on the molecular composition of *R. microplus* saliva. This is the first comprehensive proteomic study on *R. microplus* saliva. It is important to note that ticks produce minute amounts of saliva, which makes it difficult to work with as biological material, and as such it is less well characterized than salivary glands. Although some proteins reported here have already been cloned from cDNA libraries of tick tissues, they were never purified from or identified in *R. microplus* saliva.

Despite the success of tick transcriptomic studies, which provide a global view of gene expression profiles in tick salivary glands, proteomic analysis of saliva provides unique information regarding proteins that are actually secreted. In conclusion, considering the great importance of this parasite, this study improves knowledge on the tick salivary arsenal composition and gives novel insights to clarify the mechanisms associated with the tick-host relationship.

## Supporting Information

Table S1 **Tick and host proteins identified in partially engorged female saliva by 1D-LC-MS/MS.**
(DOCX)

Table S2 **Tick and host proteins identified in fully engorged female saliva by 1D-LC-MS/MS.**
(DOCX)
