# Supplementary material for: Proteomic Analysis of Cattle Tick Rhipicephalus (Boophilus) microplus Saliva: A Comparison between Partially and Fully Engorged Females
Source: PLoS One. 2014 Apr 24;9(4):e94831. doi: 10.1371/journal.pone.0094831 (PMC3998978; doi:10.1371/journal.pone.0094831)
Supplement: Table S1 — Tick and host proteins identified in partially engorged female saliva by 1D-LC-MS/MS. (DOCX) [file pone.0094831.s001.docx]

**Table S1**. Tick and host proteins identified in PEF saliva by 1D-LC-MS/MS.

| **Sample** | **Protein*^a^*** | **Accession number*^b^*** | **MW (kDa)** | **Spectral count** | **Coverage (%)** |
| --- | --- | --- | --- | --- | --- |
| PS1 | hemelipoprotein HeLP | - | 147 | 23 | 18 |
|  | vitellogenin 2 | - | 190 | 8 | 7 |
|  | vitellogenin 4 | - | 108 | 3 | 4 |
|  | hemelipoprotein HeLP 2 | - | 31 | 2 | 11 |
| PS2 | hemelipoprotein HeLP | - | 147 | 14 | 11 |
| PS3 | hemelipoprotein HeLP | - | 147 | 23 | 18 |
|  | hemelipoprotein HeLP 2 | - | 31 | 4 | 22 |
| PS4 | hemelipoprotein HeLP | - | 147 | 13 | 13 |
|  | vitellogenin 2 | - | 190 | 5 | 4 |
|  | vitellogenin 5 | - | 68 | 3 | 5 |
|  | hemelipoprotein HeLP 2 | - | 31 | 2 | 11 |
| PS5 | **serum albumin** | IPI01028455.1 | 69 | 5 | 7 |
|  | hemelipoprotein HeLP | - | 147 | 2 | 2 |
| PS6 | **serum albumin** | IPI01028455.1 | 69 | 12 | 19 |
|  | **keratin 6A-like** | IPI01002591.1 | 63 | 2 | 4 |
| PS7 | *R. microplus* serpin-17 (Rms-17) | KC990116 | 43 | 6 | 13 |
|  | secreted protein 34 | - | 37 | 2 | 10 |
| PS8 | *R. microplus* serpin-17 (Rms-17) | KC990116 | 43 | 9 | 21 |
|  | antigen 5/SCP domain-containing protein 1 | - | 46 | 3 | 9 |
| PS9 | *R. microplus* serpin-6 (Rms-6) | KC990105 | 44 | 6 | 21 |
|  | actin | AAP79880 | 42 | 4 | 15 |
|  | antigen 5/SCP domain-containing protein 1 | - | 46 | 3 | 9 |
|  | hemelipoprotein HeLP | - | 147 | 3 | 3 |
| PS10 | secreted protein 27 | - | 37 | 3 | 9 |
|  | **keratin 6A-like** | IPI01002591.1 | 63 | 2 | 4 |
| PS11 | secreted protein 19 | - | 36 | 6 | 22 |
|  | secreted protein 27 | - | 37 | 3 | 9 |
| PS12 | secreted protein 19 | - | 36 | 2 | 10 |
| PS13 | secreted protein 19 | - | 36 | 7 | 22 |
|  | lipocalin 41 | - | 16 | 2 | 22 |
|  | Kazal/SPARC domain-containing protein | - | 33 | 2 | 10 |
| PS14 | **hemoglobin subunit beta** | IPI00716455.1 | 16 | 2 | 22 |
|  | salivary lipid-interacting protein 1 | - | 20 | 2 | 16 |
| PS15 | salivary lipid-interacting protein 1 | - | 20 | 4 | 24 |
|  | **hemoglobin subunit beta** | IPI00716455.1 | 16 | 3 | 30 |
|  | Se-dependent glutathione peroxidase | - | 18 | 2 | 18 |
| PS16 | secreted protein 20 | - | 25 | 7 | 30 |
|  | salivary lipid-interacting protein 1 | - | 20 | 2 | 16 |
|  | **odorant binding protein-like** | IPI00722909.1 | 20 | 2 | 19 |
| PS17 | **allergen Bos d 2** | IPI00708946.1 | 20 | 2 | 14 |
| PS18 | **allergen Bos d 2** | IPI00708946.1 | 20 | 4 | 26 |
| PS19 | **allergen Bos d 2** | IPI00708946.1 | 20 | 3 | 21 |
|  | ML domain-containing protein 1 | - | 13 | 3 | 25 |
| PS20 | **hemoglobin subunit beta** | IPI00716455.1 | 16 | 4 | 30 |
| PS21 | **hemoglobin subunit beta** | IPI00716455.1 | 16 | 4 | 30 |
| PS22 | **hemoglobin subunit beta** | IPI00716455.1 | 16 | 9 | 54 |
| PS23 | **hemoglobin subunit beta** | IPI00716455.1 | 16 | 6 | 46 |
|  | **hemoglobin subunit alpha** | IPI00710783.2 | 15 | 3 | 25 |
| PS24 | **hemoglobin subunit beta** | IPI00716455.1 | 16 | 4 | 30 |

***^a^***Identified bovine proteins are presented in bold.

*^b^*Acession numbers for tick identified proteins were deposited as Transcriptome Shotgun Assembly project at DDBJ/EMBL/GenBank under the accessions GBBO00000000 and GBBR00000000. The versions described in this paper are the first version, GBBO01000000 and GBBR01000000, respectively
