# Supplementary material for: Proteomic Analysis of Cattle Tick Rhipicephalus (Boophilus) microplus Saliva: A Comparison between Partially and Fully Engorged Females
Source: PLoS One. 2014 Apr 24;9(4):e94831. doi: 10.1371/journal.pone.0094831 (PMC3998978; doi:10.1371/journal.pone.0094831)
Supplement: Table S2 — Tick and host proteins identified in fully engorged female saliva by 1D-LC-MS/MS. (DOCX) [file pone.0094831.s002.docx]

**Table S2**. Tick and host proteins identified in FEF saliva by 1D-LC-MS/MS.

| **Sample** | **Protein*^a^*** | **Accession number** | **MW (kDa)** | **Spectral count** | **Coverage (%)** |
| --- | --- | --- | --- | --- | --- |
| TS1 | hemelipoprotein HeLP | - | 147 | 5 | 4 |
|  | vitellogenin 2 | - | 190 | 4 | 4 |
|  | vitellogenin 4 | - | 108 | 2 | 3 |
| TS2 | hemelipoprotein HeLP | - | 147 | 19 | 16 |
| TS3 | hemelipoprotein HeLP | - | 147 | 19 | 19 |
|  | hemelipoprotein HeLP 2 | - | 31 | 4 | 27 |
|  | **keratin, type I cytoskeletal** | IPI00698285.3 | 55 | 2 | 4 |
| TS4 | **serotransferrin** | IPI00690534.1 | 78 | 11 | 19 |
|  | **serum albumin** | IPI00708398.2 | 70 | 10 | 17 |
| TS5 | **serum albumin** | IPI01028455.1 | 69 | 28 | 38 |
|  | **serum albumin** | IPI00708398.2 | 70 | 28 | 38 |
|  | **complement C3 (Fragment)** | IPI00713505.2 | 187 | 2 | 2 |
| TS6 | **serpin A3-2** | IPI00700622 | 46 | 4 | 12 |
|  | **serpin A3-2** | IPI00930024.1 | 46 | 4 | 12 |
| TS7 | secreted protein 34 | - | 37 | 2 | 10 |
| TS8 | *R. microplus* serpin-17 (RmS-17) | KC990116 | 43 | 4 | 10 |
|  | **fibrinogen gamma chain** | IPI00843209.1 | 50 | 3 | 9 |
|  | **keratin type I cytoskeletal** | IPI00721270.4 | 52 | 2 | 4 |
| TS9 | *R. microplus* serpin-3 (RmS-3) | KC990102 | 43 | 7 | 23 |
|  | **fibrinogen beta chain** | IPI00709763.5 | 56 | 6 | 16 |
|  | **hemoglobin subunit beta** | IPI00716455.1 | 16 | 3 | 22 |
| TS10 | *R. microplus* serpin-6 (RmS-6) | KC990105 | 44 | 5 | 14 |
|  | **fibrinogen beta chain** | IPI00709763.5 | 56 | 4 | 11 |
|  | **hemoglobin subunit beta** | IPI00716455.1 | 16 | 3 | 22 |
| TS11 | secreted protein 19 | - | 36 | 3 | 14 |
|  | secreted protein 27 | - | 37 | 2 | 9 |
| TS12 | **hemoglobin subunit beta** | IPI00716455.1 | 16 | 5 | 39 |
| TS13 | **hemoglobin subunit beta** | IPI00716455.1 | 16 | 29 | 56 |
|  | **hemoglobin subunit alpha** | IPI00710783.2 | 15 | 22 | 60 |

***^a^*** Identified bovine proteins are presented in bold.

*^b^*Acession numbers for tick identified proteins were deposited as Transcriptome Shotgun Assembly project at DDBJ/EMBL/GenBank under the accessions GBBO00000000 and GBBR00000000. The versions described in this paper are the first version, GBBO01000000 and GBBR01000000, respectively.
